# The Call to Strengthen the Nursing Profession

**Published:** 1918-07-27

**Authors:** 


### THE MATRONS' AND SISTERS' DEPARTMENT.

### THE CALL TO STRENGTHEN THE NURSING PROFESSION.

EVERY profession depends for its power and influence not on mere numbers, but on the creation within itself of a stable element, fitted for government. The lack of this stable element in feminine occupations has tended more than any other cause to keep women back from organising themselves. The conditions under which a woman works, and her interest in the things she does, tend, in the vast majority of cases, to take but a second place in her thoughts when once the early years of apprenticeship to her calling are over. New and overwhelming calls engross her mind. Marriage, child-bearing, home, and kindred crowd out the professional keenness with which she started on her career. This is true of so large a proportion of women that unless it be steadily kept in view in the organisation of any profession much disappointment will inevitably result. It has not been sufficiently apprehended by those who have hitherto endeavoured to help nurses to help themselves.

No one can fail to be struck by the apparent infirmity of purpose which distinguishes nurses in the things which concern their own interests. At one time, with supreme effort, nurses rally to the call of a leader. Within a short time the impulse to coalesce has faded. The flourishing little society finds itself depleted of members. Those who supported it have passed on, and the work of organisation is all to do again. It is for this reason that the nursing profession requires a permanent body like the College of Nursing amid its constant fluctuations, a stable body charged with the duty of looking after its interests. Such a body can never be adequately equipped for its tasks through voluntary subscriptions. It must not depend for the continuance of its activities on the business habits of its members, scattered as they are over the face of the world. It cannot depend for the extension of its influence on the number of members who may happen to keep in touch with it at a given moment. It must therefore be adequately endowed for its purposes, although should it fail for any reason to enlist the support of nurses as a united body it will never be able to speak with the authority needed to ensure the best results for its clients.

Regarding nurses as a stream of women merely passing through their profession, seldom consciously dedicating themselves to a life-time of nursing, but surrendering at any rate some of the best years of their life to it, it will be agreed that the period when they are most accessible to convictions about nursing matters is at the completion of their training. The most thoughtless, the least "social" in her outlook feels a thrill of professional pride when she receives the certificate which enrols her in the honoured order of nurses. Then is the time for her to mark her sense that something is due from her as well as to her, by giving her adhesion to the College of Nursing. The attainment of State registration can never alter this primary duty of the nurse to her fellows on entering her profession. She may remain a nurse for a few years only, possibly only for a few months. Still, she has tasted the dignity of her profession. She has been moulded according to its pattern. It may be she will have ample future cause to be thankful that she enrolled herself as a member of a great professional body which untiringly pursues its course in her interests, so that when, after years of oblivion, she may need to take up nursing again, there may still be a place for her, with perchance better conditions of service than she ever dreamed of in the old days.

It is very necessary that those in authority should keep before their nurses continually the imperative need in the nursing profession for the establishment of this stable element. Where so much fluctuates, where so many enter but to pass away to fresh interests, the call to build up a strong central body is very insistent. On those who object that there is no reason for them to join the College—they are engaged to be married, or they have a private income, or they are going to live at home—it may be urged that the mere fact of having taken training as a nurse entails a debt of honour. None can seriously contend that they have received nothing from their profession in the act of entering it. Their character has been enriched, their powers immeasurably extended, their value as women increased. Can they take these benefits and feel no touch of generous desire to strengthen the profession which has already done so much for them by the only act in their power? To join the College is to leave their mark even in passing through.

**NOTE.—II. "Some Hardening Influences in Training" is unavoidably held over until next issue.**

